# Green Sulfation of Arabinogalactan in the Melt of a Sulfamic Acid–Urea Mixture

**DOI:** 10.3390/polym17050642

**Published:** 2025-02-27

**Authors:** Vladimir A. Levdansky, Alexander V. Levdansky, Yuriy N. Malyar, Timur Yu. Ivanenko, Olga Yu. Fetisova, Aleksandr S. Kazachenko, Boris N. Kuznetsov

**Affiliations:** 1 Institute of Chemistry and Chemical Technology SB RAS, Federal Research Center, Krasnoyarsk Science Center SB RAS, Akademgorodok 50, Bld. 24, Krasnoyarsk 660036, Russia; vlevdanskij@mail.ru (V.A.L.); alexsander.l@mail.ru (A.V.L.); yumalyar@gmail.com (Y.N.M.); timivonk@gmail.com (T.Y.I.); fou1978@mail.ru (O.Y.F.); 2 School of Non-Ferrous Metals and Material Science, Siberian Federal University, Pr. Svobodny 79, Krasnoyarsk 660041, Russia; 3 Institute of Chemical Technologies, Reshetnev Siberian State University of Science and Technology, Mira St. 82, Krasnoyarsk 660049, Russia; 4 Professor V. F. Voino-Yasenetsky Krasnoyarsk State Medical University of the Ministry of Healthcare of the Russian Federation, St. Partizan Zheleznyak, Bld. 1, Krasnoyarsk 660022, Russia

**Keywords:** arabinogalactan, green sulfation, melt, sulfamic acid–urea mixture, sulfated arabinogalactan

## Abstract

Sulfation of arabinogalactan (AG) from larch wood (*Larix sibirica Ledeb.*) in the melt of a sulfamic acid–urea mixture has been first examined. The impact of the AG sulfation temperature on the AG sulfate yield and the sulfur content has been established. The high sulfur content (11.3–11.6%) in sulfated AG has been obtained in the temperature range of 115–120 °C for a sulfation time of 0.5 h. The process effectively prevents molecular degradation under these conditions. The incorporation of sulfate groups into the arabinogalactan structure has been confirmed by the appearance of absorption bands in the FTIR spectrum that are typical of sulfate group vibrations. The ^13^C NMR spectroscopy study has proven that the AG sulfation in the melt of a sulfamic acid–urea mixture leads to the substitution of some free hydroxyl groups for C6, C4, and C2 carbon atoms of the AG β-D-galactopyranose units. The advantage of the proposed AG sulfation method is that the reaction occurs without solvent, and the reaction time is only 0.5 h. The kinetics of the thermal decomposition of the initial AG and sulfated AG samples have been studied. It has been found that the sulfated AG samples have a lower thermal resistance than the initial AG. The kinetic analysis has revealed a decrease in the activation energy of the thermal degradation of the sulfated samples as compared to the initial AG.

## 1. Introduction

Plant polysaccharides and products of their chemical modification are increasingly used in medicine for the treatment and prevention of diseases [1,2]. Most of them are biocompatible, biodegradable, hydrophilic, well-stable, safe, and non-toxic. All this gives them a number of advantages over other synthetic polymers. Plant polysaccharides have various types of biological activity, including antivirus, antibacterial, anti-tumor, immunomodulatory, antiradiation, antioxidation, antidiabetic, anti-inflammatory, hepatoprotective, thus taking on a great therapeutic potential in medicine, food, and cosmetics [3,4,5]. However, existing studies have demonstrated that the bioactivities of polysaccharides are significantly influenced by their monosaccharide composition, glycoside bond, molecular size, branching degree, molecular conformation, and functional group, as well as other structural characteristics [6]. Thus, biopolymers are often structurally modified to improve their biological properties while generating new functional activities.

Arabinogalactan (AG) polysaccharide, the content of which in larch wood reaches 15–20 wt %, is known well and has a high application potential. AG has a wide spectrum of biological activities and exhibits many valuable characteristics: it is low-toxic, highly water-soluble, and low-viscous in concentrated liquors, can bind fat and keep moisture, and has good dispersion properties. The combination of the listed parameters augurs well for the use of AG for the treatment of humans and animals and the production of food and beauty aids [7,8]. Products of chemical modification of AG are of considerable interest to the pharmaceutical industry and medicine [9]. A technique for the chemical modification of AG that holds much promise is the introduction of sulfate groups, which leads to the production of sulfated AG derivatives with hypolipidemic and membranotropic activities [10]. Anticoagulants based on sulfated AG can be used instead of heparin in thrombolytic therapy [10,11]. Heparin obtained from raw materials of animal origin may contain pathogens dangerous to humans [12]. Therefore, the development of efficient and environmentally safe methods for the green synthesis of plant polysaccharide sulfates is gaining more attention.

The available techniques for synthesizing AG sulfates require deleterious sulfating agents and harmful solvents, including sulfuric anhydride, chlorosulfonic acid, pyridine, and N,N-dimethylformamide (DMF), or mixtures thereof [11,13,14]. In [10], it was proposed to use less aggressive and harmful reagents for AG sulfation: dry potassium persulfate in the dimethylsulfoxide (DMSO) medium. The long duration of the sulfation reaction (up to 6 h) and the difficulties of isolating and purifying AG sulfate are the disadvantages of this method of AG sulfation. In another work, sulfation of AG with ammonium sulfamate in the piperidine, 1,4-dioxane, and other inert solvents with the use of the KMnO_4_ and K_2_Cr_2_O_7_ agents or base activators (urea and thiourea) was exploited [15]. It was demonstrated that the highest (11.3 wt %) concentration of sulfur in the sulfate obtained corresponds to the process carried out in 1,4-dioxane at 90 °C for 4 h using the KMnO_4_ oxidizer. However, since the activators were applied, it was necessary to purify the obtained products by dialysis from inorganic salts.

We studied the sulfation of AG with sulfamic acid in 1,4-dioxane using urea [16]. This made it possible to replace aggressive and toxic sulfating agents (sulfuric anhydride and chlorosulfonic acid) with a stable and less toxic crystalline substance: sulfamic acid. This method allows for the synthesis of AG sulfates with a sulfur content of 11.5–12.8 wt % at a sulfation temperature of 85–90 °C for 2.5–3.0 h. However, the sulfation with sulfamic acid proceeds under heterogeneous conditions, which reduces the efficiency of the sulfation process. Also, this results in products that are heterogeneous in chemical composition. To solve this problem, we proposed to replace the toxic 1,4-dioxane solvent with DMSO [17]. DMSO dissolves AG, and sulfation occurs in homogeneous conditions. The AG sulfate with a sulfur concentration of 12.0–12.5 wt % was obtained at 85–90 °C during 2–3 h. At the same time, the high cost of the solvents and the need for recycling solvent components make it difficult to apply all of the above methods in the commercial production of AG sulfates.

The study of [18] described the sulfation of wood cellulose fibers in the melt of a sulfamic acid–urea mixture. The product with a degree of substitution (DS) of 0.68 was obtained at a temperature of 150 °C for half an hour. The absence of solvents lowers the cost of the proposed technique and ensures its safety and ecological friendliness. Nevertheless, in the absence of solvent, the mechanism of polysaccharide degradation can change, which is known [19] to affect its biological activity.

We used this approach to synthesize AG sulfate. In contrast to cellulose sulfates, which are poorly soluble in water at low degrees of substitution, initial AG and its sulfated derivatives are well soluble in aqueous systems. In addition, the methods of isolation of AG from larch wood are based on its extraction with water, whereas the manufacture of cellulose requires the use of hazardous chemicals for the wood delignification and pulp bleaching.

In this work, the optimal conditions of larch AG sulfation in the melt of a sulfamic acid–urea mixture were first established. They allow us to obtain sulfated AG with a rather high yield and sulfur content without molecular degradation. The proposed method makes it possible to modify the biopolymer rapidly (for 0.5 h) and without contamination of the resulting product with toxic solvents.

## 2. Materials and Methods

### 2.1. Materials

The raw material used in this study was Siberian larch wood (*Larix sibirica Ledeb.*) cut in May 2022 at Krasnoyarsk, Siberia. Sawdust (fraction 1–3 mm) with a moisture content of up to 5% was used to isolate AG.

### 2.2. Isolation of Arabinogalactan

Larch wood (fraction 1–3 mm, 100 g) and distilled water (1.5 L) were put into a flask (2 L) with a reflux condenser; after that, the mixture was heated at 100 °C for 2 h. Then, the heated solution was filtered from the wood, cooled to 50 °C, and evaporated with an evacuated rotary unit at 50–55 °C to 60–70 mL. The resulting aqueous AG solution was cooled down to 15–20 °C and diluted with 95% ethanol (300 mL) under stirring. The precipitated AG was separated by filtration using a water aspirator pump, Buchner funnel, and Bunsen flask in vacuum, washed on the filter with ethanol (40–50 mL), and dried at room temperature. The AG yield was 18.7% of the larch wood weight.

### 2.3. Sulfation of Arabinogalactan

Sulfamic acid (2.91 g) and urea (1.80 g) were put into a pear-shaped flask with a stirrer and heated on a LOIP LT-205a thermostat at 110 °C until the mixture melted. AG (1.60 g) isolated from larch wood was added into a flask with molten mixture; next, the flask content was subjected to stirring for 0.5 h. After that, the reaction mixture was cooled down to 15–20 °C, dissolved in a 5% aqueous sodium hydroxide solution (40–45 mL) at pH 9–10, and stirred under heating at 65–70 °C until the end of ammonia release. The resulting solution of the sodium salt of AG sulfate was dialyzed against distilled water for 24 h in a 25 mm-wide MF-8030-25 MFPI bag (Membrane Filtration Products, Inc., Seguin, TX, USA) with pores 6–8 kDa in size. The water was changed at 2 h intervals. After dialysis, an aqueous solution of AG sulfate was evaporated in a vacuum on a rotational evaporator at 50–55 °C until complete dryness was achieved. The yield of the sodium salt of AG sulfate was 1.87 g. Sulfation of AG at temperatures of 115, 120, 125, 130, and 140 °C was carried out similarly.

### 2.4. Characterization Techniques

The sulfur content in AG sulfate was found with a FlashEA™ 1112 analyzer (Thermo Fisher Scientific, Waltham, MA, USA).

The degree of substitution (DS) of the AG sulfate product was determined from the sulfur mass as follows [17]:

(1) $$DS=\frac{158\times W(S)}{A_{r}-102\times W(S)},$$

where *W(S)* is the sulfur mass fraction, *A_r_(S)* is the sulfur atomic weight, 102 is the growth of the AG unit weight caused by incorporation of a SO_3_Na group, and 158 is the AG unit average molecular weight as reported in [8,20].

FTIR spectra of the samples were recorded with the use of an IRTracer-100 FTIR spectrometer (Shimadzu, Kyoto, Japan) at wavelengths of 400–4000 cm^−1^. The results of the FTIR study were processed using the OPUS 5.0 software. The solid specimens were tableted in the KBr matrix (2 mg of a sample per 1000 mg of KBr).

Proton-decoupled ^13^C NMR spectra of AG and sulfated AG were obtained at 25 °C on a Bruker Avance III spectrometer (600 MHz (^1^H) and 155 MHz (^13^C)) in D_2_O. A recorded spectrum was subjected to processing when 4096 transients were accumulated with the 10 s relaxation delay. Two-dimensional HSQC spectra were obtained with the edited-HSQC pulse sequence from the Bruker library (hsqcedgp). Approximately 80 mg of the sample was dissolved in 0.6 mL of D_2_O for 12 h. For recording double-quantum filtered COSY spectra (DQF-COSY), 256 experiments with four scans each were performed with a delay of 6.5 s. HSQC and HMBC spectra were recorded using shape pulse sequences to water suppression with phase sensitivity using Echo/Antiecho-TPPI gradient selection, 126 experiments with 64 scans each were performed with a delay of 6.5 s.

The weighted average and number average molecular weights (Mw and Mn, respectively) and the polydispersity index (PDI)) of the synthesized samples were determined using gel permeation chromatography (GPC) on an Agilent 1260 Infinity II Multi-Detector GPC/SEC System (Agilent Technologies, Santa Clara, CA, USA) by triple detection: refractometry (RI), viscometry (VS), and light scattering (LS). Separation occurred on two PL Aquagel-OH Mixed-M columns with the 0.1 M NaNO_3_ mobile phase (250 ppm NaN_3_) in water. The columns were calibrated by polyethylene glycol standards (Agilent Technologies, Santa Clara, CA, USA). The eluent flowed at a rate of 1 mL/min. The sample volume was 100 μL. The samples were pre-dissolved in the mobile phase (5 mg/mL) and filtered on a 0.45 µm Millipore Agilent PES membrane filter. The data of this examination were collected and processed in the Agilent 2.2 GPC/SEC MDS software.

The thermogravimetric analysis (TGA) was carried out on a NETZSCH STA 449 F1 Jupiter analyzer (NETZSCH, Selb, Germany) in a corundum crucible at 30–800 °C in argon at a rate of 10 °C/min. To interpret the TGA results, the NETZSCH Proteus Thermal Analysis 5.1.0 package was applied.

## 3. Results and Discussion

### 3.1. AG Sulfation in the Melt of a Sulfamic Acid–Urea Mixture

In [18], the sulfation of cellulose with a melt of a mixture consisting of sulfamic acid and urea taken in molar ratios of 1:2, 1:3, and 1:4 was described. The resulting mixture was shown to fully pass to the liquid state at 80 °C when stirred for 30 min. Cellulose was loaded into the melt; after that, the reaction mixture was heated to 150 °C and kept for 0.5 h. Cellulose was sulfated with a 3.0- or 10.6-fold excess of sulfamic acid per one glucopyranose unit of cellulose. In the cited work [18], it was noted that a decrease in the urea content in the sulfating mixture improved the sulfation efficiency and reduced cellulose carbamatization.

**Figure 1:**
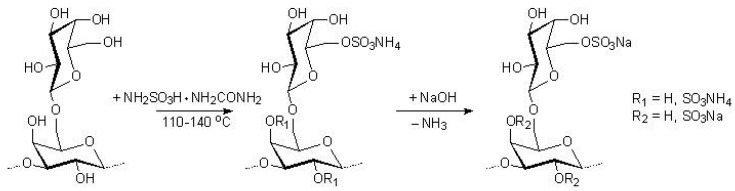
Schematic of arabinogalactan sulfation with the melt of sulfamic acid–urea mixture.

Taking into account the positive and negative aspects of cellulose sulfation using the sulfamic acid–urea melt [18], to reduce carbamatization, we sulfated AG with the sulfamic acid–urea mixture with a component ratio of 1:1. The minimum temperature at which such a sulfating mixture becomes liquid is 108–110 °C. There has been a lack of literature data on AG sulfation with the molten sulfamic acid–urea mixture. Therefore, the AG sulfation reaction was carried out with a melt of sulfamic acid–urea mixture at temperatures of 110, 115, 120, 125, 130, and 140 °C for 0.5 h. The AG sulfate sodium salt was obtained (see Figure 1).

The data on the effect of the temperature of the molten sulfamic acid–urea mixture on the AG sulfate yield and content of sulfur in the reaction products are given in Table 1.

According to the results listed in Table 1, a high sulfur content (11.3–11.6 wt %) and a yield of 2.03–2.11 g were achieved after AG sulfation at a temperature of 115–120 °C for 0.5 h. As the temperature is enhanced to 125 °C, the sulfur content drops to 10.2 wt % and the yield of sulfated AG, to 1.95 g. At the subsequent sulfation temperature growth to 140 °C, the sulfated AG yield lowers to 1.79 g and the sulfur content, to 7.7 wt %. This temperature intensifies degradation reactions and the produced AG sulfate turns brown.

The sulfur content in the sulfated AG samples (11.3–11.6%) synthesized in the melt of a sulfamic acid–urea mixture was identical to the sulfur content obtained by AG sulfation with ammonium sulfamate/KMnO_4_ in the 1,4-dioxane medium (11.3 wt %) [15], AG sulfation in the sulfamic acid–urea mixture using the 1,4-dioxane (11.5–11.8%) [16], and DMSO (12.0–12.5 wt %) media [17]. The sulfur content in the sample obtained by the sulfation of AG with potassium persulfate in the DMSO medium was slightly higher (14.2 wt %) [10], which is probably due to the longer reaction time (6 h). This shows the sufficient efficiency of the new sulfation method. However, this method has potential limitations. Thus, it is necessary to ensure thorough mixing and uniform heating of the reaction mixture to avoid its foaming during reaction scaling.

### 3.2. Characterization of Arabinogalactan and Sulfated Arabinogalactan

The structural study of larch wood AG was carried out using FTIR and ^13^C NMR spectroscopy.

**Figure 2:**
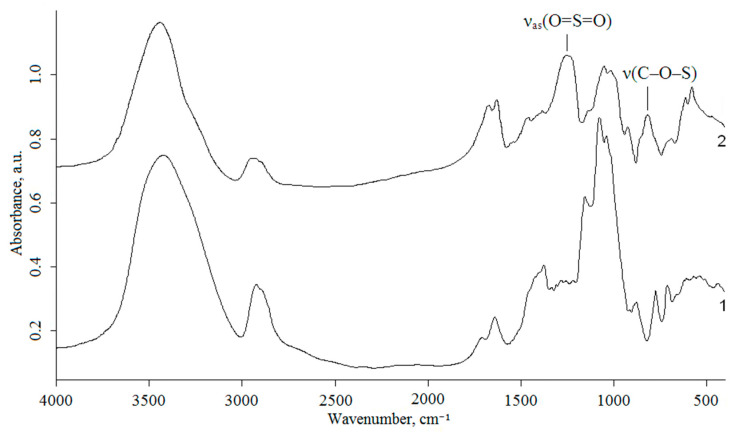
FTIR spectra of AG (1) and AG sulfate with a sulfur content of 11.6 wt % (2).

In the AG FTIR spectrum (Figure 2), one can distinguish a pronounced band near 3423 cm^−1^, which reflects stretching vibrations of the hydroxyl groups associated with hydrogen bonds. The bands at 2923 cm^−1^ correspond to stretching vibrations of CH_2_– and CH– groups of carbohydrate units. In addition, the medium-intensity band around 1641 cm^−1^ belongs to water molecules associated with the biopolymer matrix. The range of 1200–1000 cm^−1^ is related to ring vibrations superimposed by stretching vibrations of the C–OH side groups and the vibration of the C–O–C glycosidic bond. Thus, in this region, the absorption band at 1078 cm^−1^ dominates, which is characteristic of a galactopyranose backbone in arabino-3-6-galactan [21]. The band at 878 cm^−1^ is indicative of the β-D-glucosidic bond of AG pyranose rings, while the absorption bands at 775 and 713 cm^−1^ are related to a furanose ring skeleton [22].

The sulfated AG FTIR spectra presented in Figure 2 contain, in contrast to the initial AG spectra, absorption bands at 818 and 1257 cm^−1^, which is evidence for the incorporation of sulfate groups into the AG structure. The pronounced band at 1257 cm^−1^ is indicative of asymmetric stretching vibrations υ_as_(O=S=O). The absorption band in the region of 816–820 cm^−1^ is attributed to stretching vibrations υ(C–O–S) of the sulfated AG sodium salt [23]. In the FTIR spectrum of the initial AG, there is a high-intensity band at 3423 cm^−1^ brought in correspondence to stretching vibrations of hydrogen-bonded OH groups. However, the intensity of this band in the AG sulfate spectrum is lower, and it is shifted toward higher frequencies (3443 cm^−1^). Similarly, for the absorption band of in-plane bending vibrations of OH groups, one can observe the intensity drop and shift from 1376 to 1385 cm^−1^.

**Figure 3:**
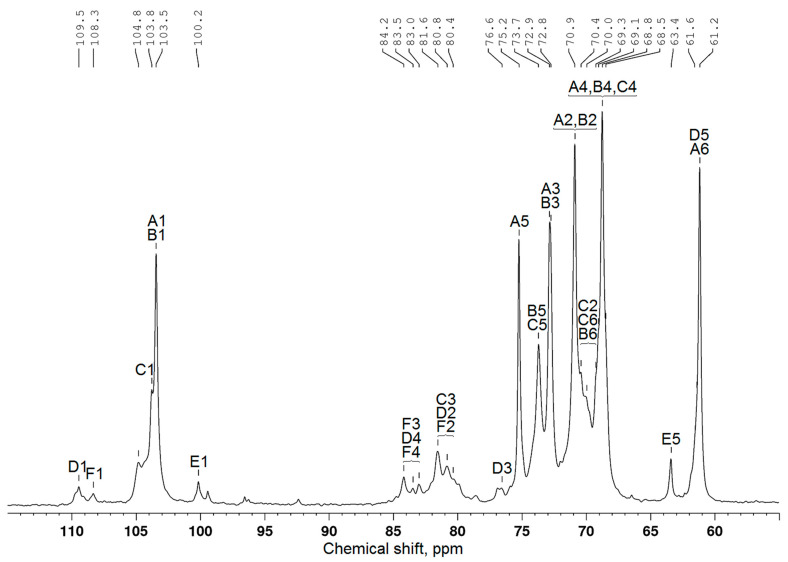
^13^C NMR spectrum of arabinogalactan.

**Figure 4:**
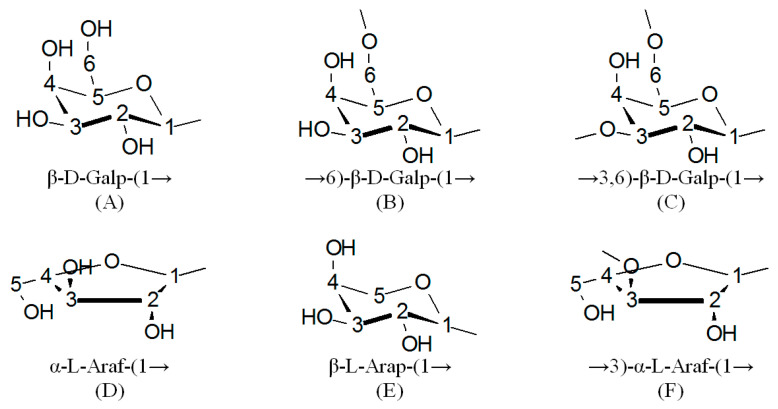
Main structural units of AG macromolecule.

**Figure 5:**
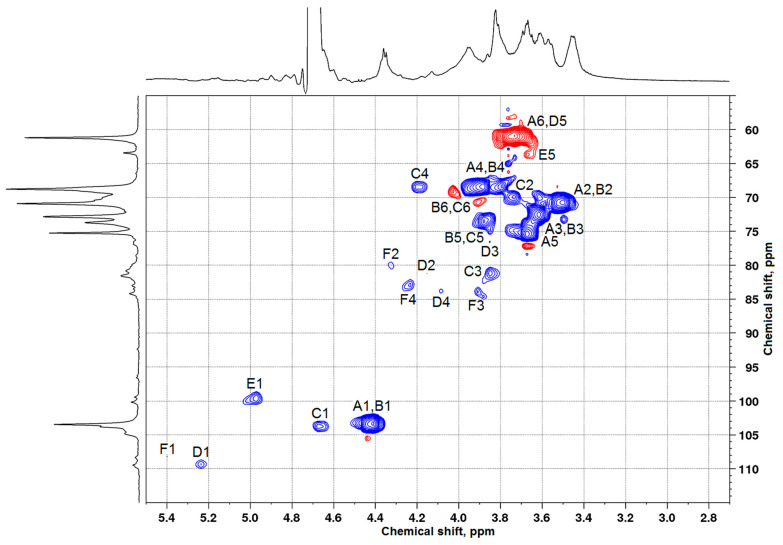
HSQC spectrum of arabinogalactan. The red color shows the proton-carbon correlations in –CH_2_– groups while the blue color shows the proton-carbon correlations in –CH– and –CH_3_ groups.

As is well known, in ^13^C NMR spectra, there are chemical shifts of C1–C6 carbon atoms in the terminal AG β-galactopyranose units at 103–104, 70–71, 72–73, 68–69, 75–76, and 61–62 ppm, respectively [7]. The values of these shifts of carbon atoms of the galactopyranose and arabinose remaining units were also determined [24]. According to the data presented in Figure 3, the ^13^C NMR spectrum of larch wood AG contains signals from all carbon atoms (Table 2) typical of galactopyranose units (Figure 4). It is noteworthy that, since peaks overlap, the examination with the use of the ^13^C chemical shifts is insufficient to attribute them with confidence to certain carbon atoms. For this reason, the data were obtained, in part, using the HSQC (Figure 5), COSY, and HMBC spectra (Figures S1 and S2 in Supplementary Materials). The correlation peaks in the HSQC spectra identified using the literature data [9,25,26,27] are given in Table 3.

It is known [20] that the structure of larch wood AG is strongly branched. It was shown in [28] that the main chain involves 3,6-di-O-substituted β-D-galactopyranose fragments, whereas side chains contain 6-O-substituted β-D-galactopyranose and terminal β-Dgalactopyranose fragments. Arabinose fragments in the AG macromolecule represent side chains including 3-O-substituted L-arabinofuranose and terminal β-L-arabinopyranose and α-L-arabinofuranose fragments.

**Figure 6:**
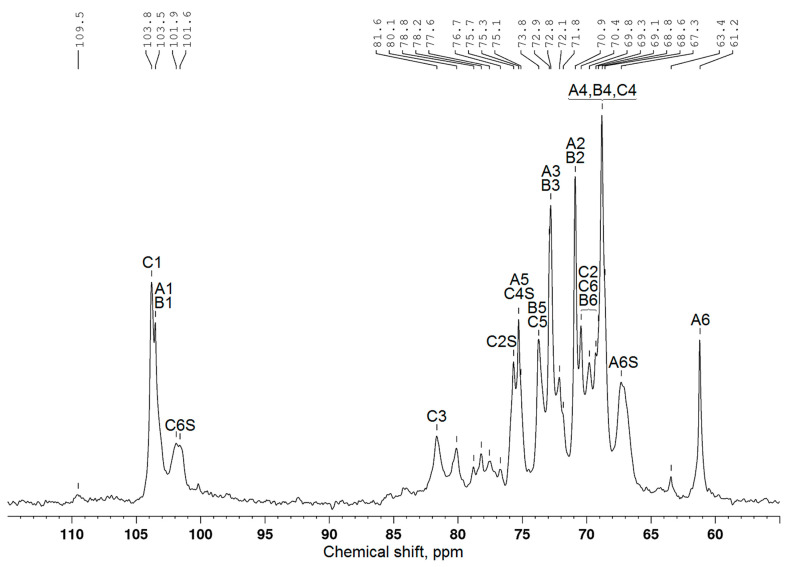
^13^C NMR spectrum of the sulfated AG sample (a sulfur content of 11.6 wt %) obtained in the melt of a sulfamic acid–urea mixture.

**Figure 7:**
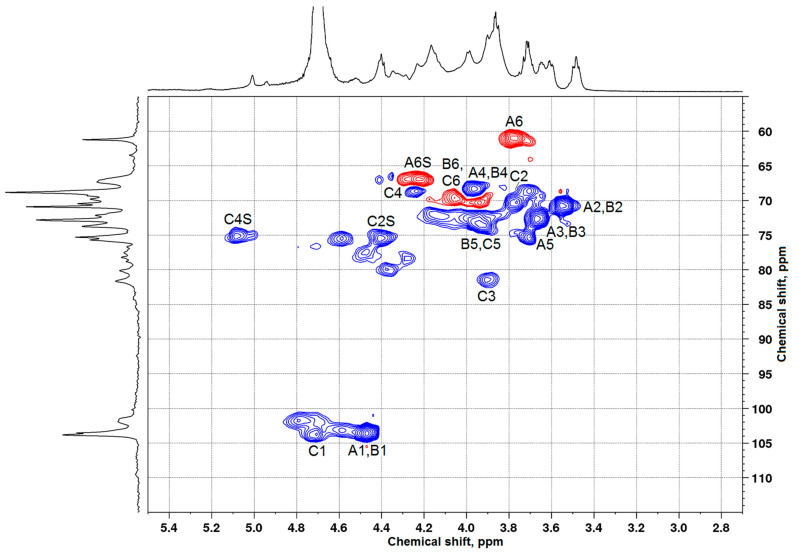
HSQC spectrum of the sulfated AG sample (a sulfur content of 11.6 wt %) obtained in the melt of a sulfamic acid–urea mixture. The red color shows the proton-carbon correlations in –CH_2_– groups while the blue color shows the proton-carbon correlations in –CH– and –CH_3_ groups.

The authors of [10,29] studied the sulfation of larch wood AG with the SO_3_–DMF complex and potassium persulfate in the DMSO medium. They demonstrated that the introduction of sulfate groups causes a downfield shift of signals from the carbon atoms bearing a sulfate group in the ^13^C NMR spectrum, while the signals of neighboring carbon atoms shifted upfield. The analogous chemical shift variations were disclosed in the comparison of the ^13^C NMR spectra of the initial and sulfated AG samples synthesized from the melt of a sulfamic acid–urea mixture. Taking these data into account and using the ^13^C NMR (Figure 6), HSQC (Figure 7), COSY, and HMBC (Figures S3 and S4 in Supplementary Materials) spectra, the presence and positioning of sulfate groups in the AG sulfate can be determined.

Along with the signals of carbon atoms of the initial β-D-galactopyranose units, the AG sulfate ^13^C NMR spectrum (Figure 6) contains additional signals corresponding to carbon atoms (Table 4). The HSQC spectrum of AG sulfate also shows the appearance of new ^1^H–^13^C cross-peaks (Table 5). Consequently, the structure of the AG macromolecule experiences changes under sulfation.

A new signal at 67.3 ppm belongs to C6 atoms corresponding to the sulfate group in the terminal β-D-galactopyranose units. This fact was confirmed by the signal weakening at 61.2 ppm for the C6 atoms corresponding to unsubstituted primary hydroxyl groups of these units, as well as a decrease in the intensity of the signal of neighboring C5 carbon atoms at 75.3 ppm. In other words, as a result of the electronic effect caused by the addition of an electron-withdrawing sulfate group instead of a hydroxyl group, the signal of C6 atoms shifted by 6.1 ppm downfield, and the signal of neighboring C5 atoms shifted upfield. The new location of the C5 signal was identified as 72.1 ppm, although it can be overlapped by the signals belonging to the C5 atoms of the side and main β-D-galactopyranose units [7,30,31].

In addition, hydroxyl groups at the C4 and C2 carbon atoms of the β-D-galactopyranose units of the main chain can enter the sulfation reaction. A whole group of new signals in the region of 75.1–75.7 ppm can be assigned to C4 and C2 atoms associated with sulfate groups. At the same time, the chemical shifts of C4 atoms of the β-D-galactopyranose units that do not contain sulfate groups are 68.8 ppm, and the chemical shifts of C2 atoms remain in the range of 70.4–70.9 ppm. The chemical shift variation under the action of sulfation of hydroxyl groups of neighboring carbon atoms can be seen for the C1 atoms of the β-D-galactopyranose units as well. The signals from the C1 atoms at 103.5–103.8 ppm partially shifted upfield (β-effect) and are assigned in the AG sulfate spectrum to the peaks around 101.6–103.2 ppm.

Note that there are difficulties with the interpretation of some signals in the AG sulfate NMR spectra. This is caused by the low dispersion of signals in the region of 65/3.50–85/4.50 ppm and the presence of very weak signals from carbon atoms of the arabinose units. According to [20], the ratio of Gal/Ara between the galactose and arabinose units in the Siberian larch AG samples is 1/9.5–11.7.

It was shown, by comparing the ^13^C NMR data on the sulfated AG samples synthesized by the proposed method with the results reported in [17], that sulfation in the melt results only in partial substitution of hydroxyl groups for C6 atoms of the terminal β-D-galactopyranose units, while sulfation in the DMSO solution results in predominant substitution of these hydroxyl groups.

**Figure 8:**
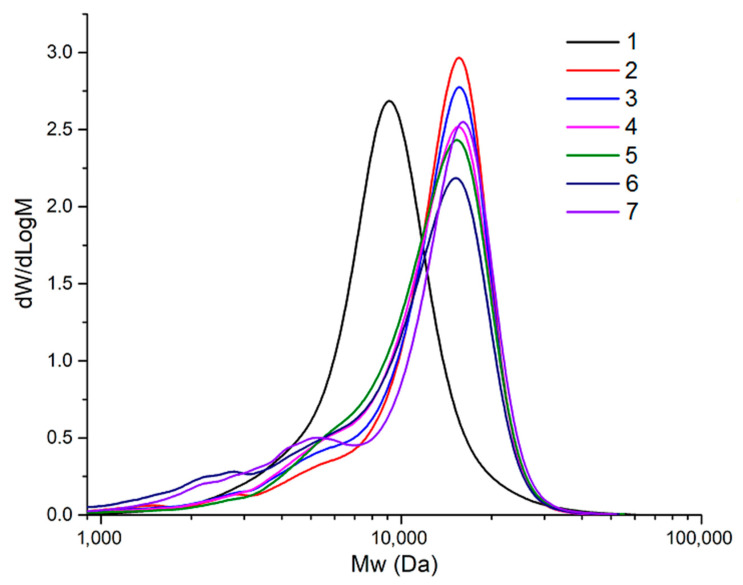
Molecular weight distributions for initial AG (1) and its sulfated derivatives obtained at temperatures of 110 (2), 115 (3), 120 (4), 125 (5), 130 (6), and 140 °C (7).

The molecular weight distribution (MWD) of polymers, especially in the processes of their chemical modification, is a critically important parameter. Features of changes in the MWD of the AG samples at different temperatures under sulfation with the melt of sulfamic acid and urea were studied by the GPC technique. Figure 8 presents the plotted molecular weight distributions for the initial and sulfated AG samples.

It was found that initial AG is characterized by a narrow MWD with a PDI of 1.27 and Mw = 9527 Da (Table 6). The sulfated AG obtained at a temperature of 110 °C has an increased Mw (13,516 Da). The MWD curve for this sample is also characterized by a monomodal distribution with a PDI increased to 1.46, which may indicate the uniform addition of sulfate groups to the arabinogalactan molecule during this process.

When the sulfation temperature is raised to 115 °C, the main peak position in the MWD of the product is maintained, but the proportion of low-molecular-weight fractions increases, which leads to a slight decrease in the values of the number-average and weight-average molecular weights of 8922 and 13,198 Da, respectively, while maintaining polydispersity. Taking into account the increase in the degree of substitution to 0.91, it can be assumed that the increase in molecular weight is due to the inclusion of additional sulfate groups in molecules with a mass of up to 8 kDa. With an increase in sulfation temperature to 125 °C, this trend continues: the proportion of highly substituted oligomeric products increases, some of which are formed in side processes of depolymerization of the initial AG chains. As the temperature increases to 130 °C, the MWD of the products undergoes stronger changes, confirming the intensification of the AG depolymerization reactions: a further decrease in the proportion of high molecular weight fractions occurs; a larger number of oligomeric products are formed; some of which are removed during dialysis, which leads to a decrease in the sulfated AG yield and degree of sulfation (Table 1).

Deterioration of the sulfated AG molecule stability can also be associated with an increase in the degree of substitution. A large number of sulfate groups on the polymer surface leads to mutual electrostatic repulsion, which, in turn, causes unwinding of the AG polymer chain and an increase in the likelihood of breaking the glycosidic bonds of the main galactose chain [32,33]. These processes become most obvious at temperatures of 130 and 140 °C. First, there is a sudden increase in polydispersity to 1.91 caused by an increase in the proportion of oligomeric depolymerization products; after that, it decreases to 1.68 with an increase in the Mw value to 12,909 Da.

### 3.3. Thermochemical Properties of Arabinogalactan and Arabinogalactan Sulfates

**Figure 9:**
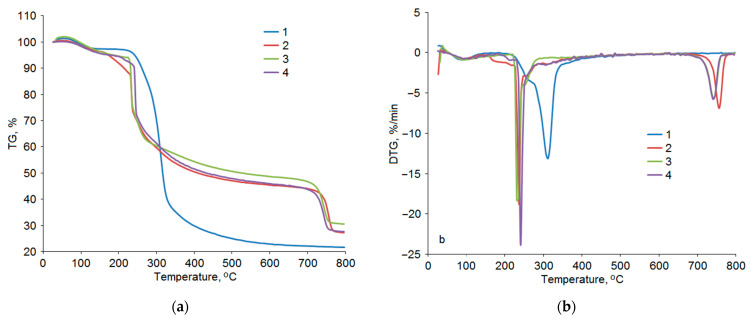
TG (**a**) and DTG (**b**) curves of thermal decomposition of initial AG (1) and sulfated AG obtained at temperatures of 110 (2), 125 (3), and 140 °C (4).

Figure 9 shows the thermogravimetry (TG) and differential thermogravimetry (DTG) curves of the samples of initial and sulfated AG obtained at temperatures of 110, 125, and 140 °C.

It is a common fact that the TG and DTG curve type is governed by many factors: heterogeneity of thermal decomposition, chemical reactions, conformational and phase transitions, etc. [34]. Thermal decomposition will apparently involve reactions of thermal degradation of AG and its derivatives with the simultaneous destruction of glycosidic bonds. Thermal stability is a key property of AG, which determines, to a great extent, the application potential of this polysaccharide.

**Figure 10:**
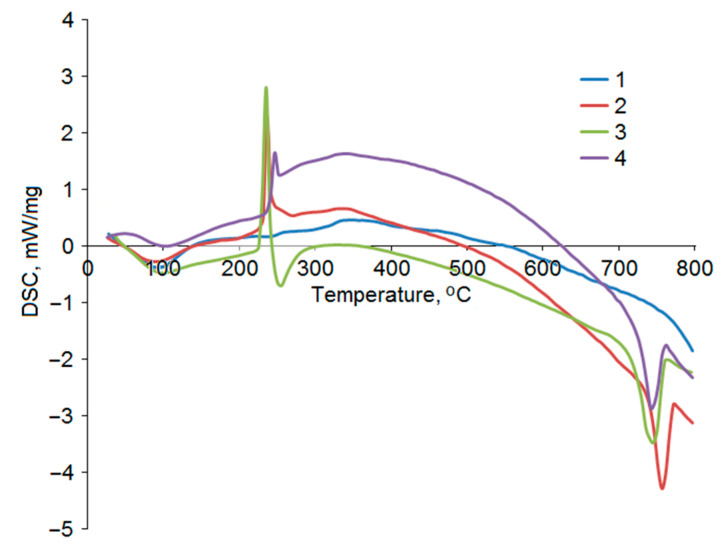
DSC curves of thermal decomposition of initial AG (1) and sulfated AG obtained at temperatures of 110 (2), 125 (3), and 140 °C (4).

The thermal decomposition of AG at 30–800 °C includes three stages. First, at 90 °C, AG starts to lose crystallization water and, next, sorbed water. At a temperature of 130 °C, the weight loss amounts to 2.3 wt %. On the DSC curve (Figure 10), this temperature corresponds to the characteristic endo effect. Thus, desorption of bound water occurs up to 130–140 °C. This fact can be explained by the difficulty of breaking hydrogen bonds between water molecules and polar functional groups of polysaccharides.

The subsequent temperature growth to 230 °C barely affects the AG structure (the weight loss in the range of 130–230 °C was 1.4 wt %). The absence of thermal effects in the DSC curve (Figure 10) in the studied temperature range confirms this assumption.

AG decomposes mainly at the second thermolysis stage, i.e., at 230–500 °C. Here, the sample loses the main part of its weight: 72 wt %. The DTG curve of initial AG is characterized by a single peak with a shoulder at 250 °C and a maximum at 312 °C. The weight loss was mainly due to the destruction of glucosidic bonds, as well as aromatization and depolymerization of the monosaccharide components of AG [35].

The third thermolysis stage (500–800 °C) results in a minor weight loss, 3.2 wt %, which can be attributed to the aromatization of the AG structure, causing coke residue formation [36].

The sulfation of AG significantly changes the nature of its thermal decomposition (Figure 9b). The temperature at the beginning of the main decomposition barely changed and amounted to 226 °C for the sulfated samples. The main decomposition of the samples was completed at 300 °C. The DTG curves of sulfated AG at this stage of decomposition are characterized by a shift in the temperature corresponding to the fastest weight loss in the series 237, 230, and 241 °C for the samples obtained at 110, 125, and 140 °C, respectively. Moreover, the decomposition rate for the sulfated samples at this heating stage was much higher than the initial AG decomposition rate. The sulfated sample obtained at 140 °C showed a maximum weight loss rate of 24%/min, whereas initial AG degrades at a rate of 13%/min. Distinct exo-effects associated with dehydrogenation, decarboxylation, and decarbonylation reactions can be seen in the DSC curves in the main period of decomposition (Figure 10) [37].

A sharp spike in weight loss was noted after 650 °C in the TG and DTG curves of the sulfated AG samples. In the temperature range of 650–760 °C, the samples obtained at 110, 125, and 140 °C lost 14.8, 16.9, and 17.0%, respectively. The high yield of carbon residue in sulfated AG obtained at 125 °C is associated with the highest sulfur content among the studied samples, since it is known [38] that sulfate groups act as fire retardants. The DSC analysis of this region shows distinct endo-effects, which, among other things, may indicate melting.

The results of the TGA study helped in the Coats—Redfern calculation of the kinetics of the main stage of decomposition of the sample [39]. The decomposition of polysaccharides is described, as a rule, using a first-order reaction [40,41,42]. The linear regression equation for calculating activation energy *E* of the process was proposed to be used in describing the thermal decomposition process:

(2) $$ln\left[-\frac{ln\left(1-\alpha \right)}{T^{2}}\right]=-\left(\frac{E}{RT}\right)+\left(\frac{AR}{\beta E}\right)\times \left(1-\frac{2RT}{E}\right),\;\mathrm{at}\;(n=1)$$

Here, *α* is the degree of thermal transformation of a substance, *β* is the heating rate, *T* is the temperature, *n* is the reaction order, *R* is the universal gas constant, and *A* is the pre-exponential factor. Conventionally, we have *RT*/*E* << 1 and 1 − 2*RT*/*E* ≈ 1. When interpreting the experimental data, the reaction order was taken to be *n* = 1. In cases where the choice of the *n* value is correct, the dependence of *ln*[−*ln*(1 − *α*)/*T*^2^] on the reciprocal temperature is approximated by a linear function with the maximum squared correlation coefficient (*R*^2^). The straight slope is proportional to activation energy *E*.

The activation energy that is determined in this manner is called the effective energy. The effective activation energy of thermal decomposition is one of the crucial indicators of the resistance of natural and synthetic polymers against high temperatures. In addition, the activation energy is used to study mechanisms of thermal destruction, aging, and stabilization of polymeric materials.

**Figure 11:**
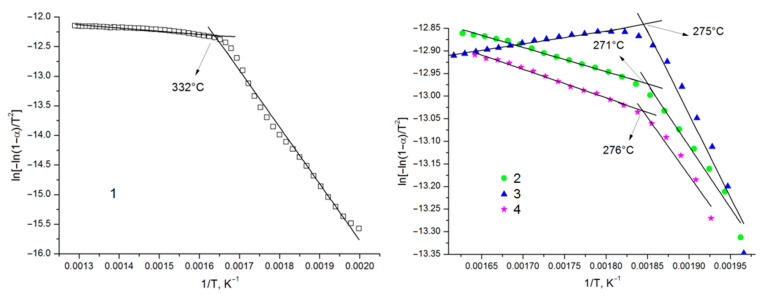
Coats—Redfern linearization of the TGA results. AG (1) and sulfated AG obtained at temperatures of 110 (2), 125 (3), and 140 °C (4).

Figure 11 shows an example of a graphical determination of the effective activation energies of the decomposition of AG and its sulfated derivatives.

The process of the main thermal destruction of the samples (Figure 11) can be divided into two linear temperature intervals, which correspond to the fastest thermal destruction and coking residue formation. The inflection point (Figure 11) corresponds to the temperature at which the mechanism of thermal destruction changes. As can be seen from Figure 11, the temperature at which the main decomposition of AG is completed is much higher (332 °C) than that for sulfated AG samples.

Table 7 gives the effective activation energies of decomposition of the investigated samples obtained using the plots. According to the table, the kinetic parameter values obtained correlate linearly, since almost all of them had a correlation coefficient of higher than 0.9.

The activation energy represents a key kinetic parameter indicating the minimum energy needed for cleaving a chemical bond. Thermal destruction of the AG derivatives was characterized by a significant decrease in activation energy compared to the initial AG. These data indicate significant disordering of the AG structure during modification.

A similar effect was observed by the authors [38] during the thermolysis of sulfated cellulose. For example, sulfated pine cellulose samples showed higher thermal degradation rates compared to the original ones, which correlated well with their higher degree of sulfation and hence lower activation energy (*E*) and accelerated degradation rates [43,44]. Cellulose nanocrystals (CNCs) obtained from bagasse and eucalyptus using concentrated sulfuric acid also showed onset of degradation at 204 °C [45] and 222 °C [46], respectively. Such low onsets were attributed to the introduction of sulfate groups into CNCs during sulfuric acid hydrolysis. Sulfate ester groups are dehydration catalysts and have been shown to reduce *E* and facilitate the thermal degradation of nanocellulose material.

A decrease in the thermal stability and, consequently, the activation energy of sulfated arabinogalactan occurs as a result of several factors. Firstly, sulfation of arabinogalactan ensures amorphization of its structure, which is confirmed by the results of X-ray diffraction analysis [15]. Loose packing promotes a change in the conformation of macromolecules, which subsequently facilitates the process of thermal decomposition of polymers.

Secondly, the number of hydroxyl groups in sulfated AG samples is significantly reduced according to the FTIR spectroscopy data, which destabilizes intra- and intermolecular hydrogen bonds. During sulfation, the available hydroxyl groups are replaced by sulfate groups, which are more easily broken down during thermolysis [38,47]. A slight increase in the activation energy of sulfated AG obtained at 125 °C can be attributed to the large number of sulfate groups on the polymer surface. An increase in the concentration of sulfate groups on the AG surface leads to mutual electrostatic repulsion, which, in turn, causes unwinding of the AG polymer chain and an increase in the probability of breaking the glycosidic bonds of the galactose backbone [32,33].

## 4. Conclusions

It was first proposed that AG sulfation be performed in the melting of a sulfamic acid and urea equimolar mixture. The method developed for synthesizing AG sulfates eliminates the need for harmful solvents (pyridine, DMF, and 1,4-dioxane) and is shorter in time, simpler, and environmentally safe compared to known methods of AG sulfation. It was established that, to obtain AG sulfates with a high sulfur content (11.3–11.6 wt %), the sulfation process should be carried out at a temperature of 115–120 °C for up to 30 min. Under these conditions, the molecular weight of AG increases proportionally to the number of included sulfate groups up to 13.2 kDa without side reactions of molecular degradation of the main chain. The presence of sulfate groups in the synthesized AG sulfates was verified by the FTIR examination. According to the results of the ^13^C NMR spectroscopy study, sulfate groups are located at the C6, C4, and C2 carbon atoms of the β-D-galactopyranose units of sulfated AG. The results of the kinetic study of the thermal degradation of initial AG and sulfated AG samples were presented. The values of the activation energy of the main identified thermal degradation stages for both the initial and sulfated AG samples were determined from the TG data using the non-isoconversional integral method. It was found that the incorporation of sulfate groups into the AG structure reduces the activation energy of the thermal decomposition main stage of sulfated AG due to the significant disordering of its structure.

## Figures and Tables

**Table 1:** Effect of the temperature of AG sulfation with the melt of a sulfamic acid–urea mixture on the AG sulfate yield and sulfur content.

N	Temperature (°C)	Time (h)	Content of Sulfur (wt %)	DS	Yield * (g)
1	110	0.5	7.9	0.52	1.87
2	115	0.5	11.6	0.91	2.11
3	120	0.5	11.3	0.89	2.03
4	125	0.5	10.2	0.75	1.95
5	130	0.5	8.5	0.58	1.87
6	140	0.5	7.7	0.50	1.79

* 1.60 g of AG was sulfated in all the experiments.

**Table 2:** Assignment of signals in the initial AG ^13^C NMR spectrum to carbon atoms.

Structural Unit	Chemical Shift (ppm)					
	C1	C2	C3	C4	C5	C6
β-D-Galp-(1→(A)	103.5	70.9	72.9	68.8	75.2	61.2
→6)-β-D-Galp-(1→(B)	103.5	70.9	72.8	68.8	73.7	69.3
→3,6)-β-D-Galp-(1→(C)	103.8	70.4	81.6	68.8	73.7	70.0
α-L-Araf-(1→(D)	109.5	80.8	76.6	83.5	61.6	–
β-L-Arap-(1→(E)	100.2	68.5	68.9 *	69.1	63.4	–
→3)-α-L-Araf-(1→(F)	108.3	80.4	84.2	83.0	61.6	–

* Some of the minor arabinose peaks were assigned according to studies [7,24].

**Table 3:** Assignment of ^1^H–^13^C cross signals in the HSQC spectrum of arabinogalactan.

Structural Unit	Chemical Shift (ppm)					
	C1/H1	C2/H2	C3/H3	C4/H4	C5/H5	C6/H6
β-D-Galp-(1→(A)	103.4/4.42	70.8/3.51	72.6/3.62	68.5/3.90	75.4/3.68	61.0/3.74
→6)-β-D-Galp-(1→(B)	103.4/4.42	70.8/3.51	72.6/3.62	68.5/3.90	73.6/3.88	69.1/4.03
→3,6)-β-D-Galp-(1→(C)	103.7/4.68	70.0/3.74	81.3/3.86	68.5/4.19	73.6/3.88	70.8/3.91
α-L-Araf-(1→(D)	109.3/5.24	81.2/4.15	76.6/3.86	83.9/4.09	61.0/3.74	–/–
β-L-Arap-(1→(E)	99.5/4.97	n.d. *	n.d.	n.d.	63.7/3.68	–/–
→3)-α-L-Araf-(1→(F)	108.2/5.40	80.0/4.33	84.0/3.92	82.9/4.24	n.d.	–/–

* n.d. means not determined.

**Table 4:** Assignment of signals in the AG sulfate ^13^C NMR spectrum to carbon atoms.

Structural Unit	Chemical Shift (ppm)					
	C1	C2	C3	C4	C5	C6
β-D-Galp-(1→(A)	103.5	70.9	72.9	68.8	75.3	61.2
Sulfated A (AS)	n.d. *	n.d.	n.d.	n.d.	72.1	67.3
→6)-β-D-Galp-(1→(B)	103.5	70.9	72.8	68.8	73.8	69.3
Sulfated B (BS)	n.d.	n.d.	n.d.	n.d.	n.d.	n.d.
→3,6)-β-D-Galp-(1→(C)	103.8	70.4	81.6	68.8	73.8	69.8
Sulfated C (CS)	101.6	75.7	n.d.	75.1	n.d.	n.d.

* n.d. means not determined.

**Table 5:** Assignment of ^1^H–^13^C cross signals in the HSQC spectrum of AG sulfate, obtained in the melt of a sulfamic acid–urea mixture.

Structural Unit	Chemical Shift (ppm)					
	C1/H1	C2/H2	C3/H3	C4/H4	C5/H5	C6/H6
β-D-Galp-(1→(A)	103.6/4.47	70.8/3.56	72.7/3.68	68.3/3.97	75.4/3.71	61.1/3.77
Sulfated A (AS)	103.2/4.59	n.d. *	n.d.	n.d.	72.1/4.15	67.0/4.23
→6)-β-D-Galp-(1→(B)	103.6/4.47	70.8/3.56	72.7/3.68	68.3/3.97	73.5/3.94	69.7/4.06
Sulfated B (BS)	n.d.	n.d.	n.d.	n.d.	n.d.	n.d.
→3,6)-β-D-Galp-(1→(C)	103.7/4.71	70.3/3.77	81.4/3.91	68.6/4.19	73.5/3.94	70.3/3.94
Sulfated C (CS)	101.8/4.80	75.5/4.41	n.d.	75.2/5.08	n.d.	n.d.

* n.d. means not determined.

**Table 6:** Molecular weights of initial AG (1) and sulfated AG obtained at temperatures of 110 (2), 115 (3), 120 (4), 125 (5), 130 (6), and 140 °C (7).

Sample	Mn, Da	Mw, Da	PDI
AG	7505	9527	1.27
sulfated AG obtained at 110 °C	9229	13,516	1.46
sulfated AG obtained at 115 °C	8922	13,198	1.48
sulfated AG obtained at 120 °C	8786	12,890	1.47
sulfated AG obtained at 125 °C	9315	12,983	1.39
sulfated AG obtained at 130 °C	6148	11,766	1.91
sulfated AG obtained at 140 °C	7666	12,909	1.68

**Table 7:** Kinetic parameters of thermal decomposition of initial AG (1) and its sulfated derivatives obtained at temperatures of 110 (2), 125 (3), and 140 °C (4).

Sample	Temperature Range, °C	Equation of the Kinetic Curve	Activation Energy *E* (kJ/mol)	Correlation Factor *R*^2^
AG	227–327 332–502	*y* = −9580.2*x* + 3.3848 *y* = −536.92*x* − 11.435	79.6 4.5	0.9894 0.9737
sulfated AG obtained at 110 °C	236–266 271–341	*y* = −2732.3*x* − 7.9194 *y* = −535.5*x* − 11.982	22.7 4.5	0.9725 0.9880
sulfated AG obtained at 125 °C	235–270 275–350	*y* = −3672.4*x* − 6.0628 *y* = −282.7*x* − 13.365	30.5 2.4	0.9492 0.9775
sulfated AG obtained at 140 °C	246–271 276–336	*y* = −2567*x* − 8.2984 *y* = −630.7*x* − 11.869	21.3 5.2	0.9541 0.9940

## Data Availability

All data generated during this study are included in the article.

## Supplementary Materials

The following supporting information can be downloaded at: https://www.mdpi.com/article/10.3390/polym17050642/s1, Figure S1: COSY spectrum of arabinogalactan isolated from *Larix sibirica* wood; Figure S2: HMBC spectrum of arabinogalactan isolated from *Larix sibirica* wood; Figure S3: COSY spectrum of sulfated arabinogalactan sample (sulfur content 11.6 wt %) obtained in the melt of a sulfamic acid–urea mixture; Figure S4: HMBC spectrum of sulfated arabinogalactan sample (sulfur content 11.6 wt %) obtained in the melt of a sulfamic acid–urea mixture.
