# Supplementary material for: Green Sulfation of Arabinogalactan in the Melt of a Sulfamic Acid–Urea Mixture
Source: Polymers (Basel). 2025 Feb 27;17(5):642. doi: 10.3390/polym17050642 (PMC11902456; doi:10.3390/polym17050642)
Supplement: Supplementary file 1 [file polymers-17-00642-s001.zip › polymers-3445999-supplementary.pdf]

Supplementary Materials

Green Sulfation of Arabinogalactan in the Melt of a Sulfamic Acid–Urea Mixture

Vladimir A. Levdansky [1], Alexander V. Levdansky [1], Yuriy N. Malyar [1,2], Timur Yu. Ivanenko [1], Olga Yu. Fetisova [1], Aleksandr S. Kazachenko [1,2,3,*] and Boris N. Kuznetsov [1,2]

[1] Institute of Chemistry and Chemical Technology, Krasnoyarsk Science Center, Siberian Branch, Russian Academy of Sciences, Akademgorodok 50, bld. 24, Krasnoyarsk, 660036 Russia

[2] School of Non-Ferrous Metals and Materials Science, Siberian Federal University, pr. Svobodny 79, Krasnoyarsk, 660041 Russia

[3] Institute of Chemical Technologies, Reshetnev Siberian State University of Science and Technology, Mira st. 82? Krasnoyarsk, Russia

* Corresponding Author: A.S. Kazachenko, e-mail: leo_lion_leo@mail.ru

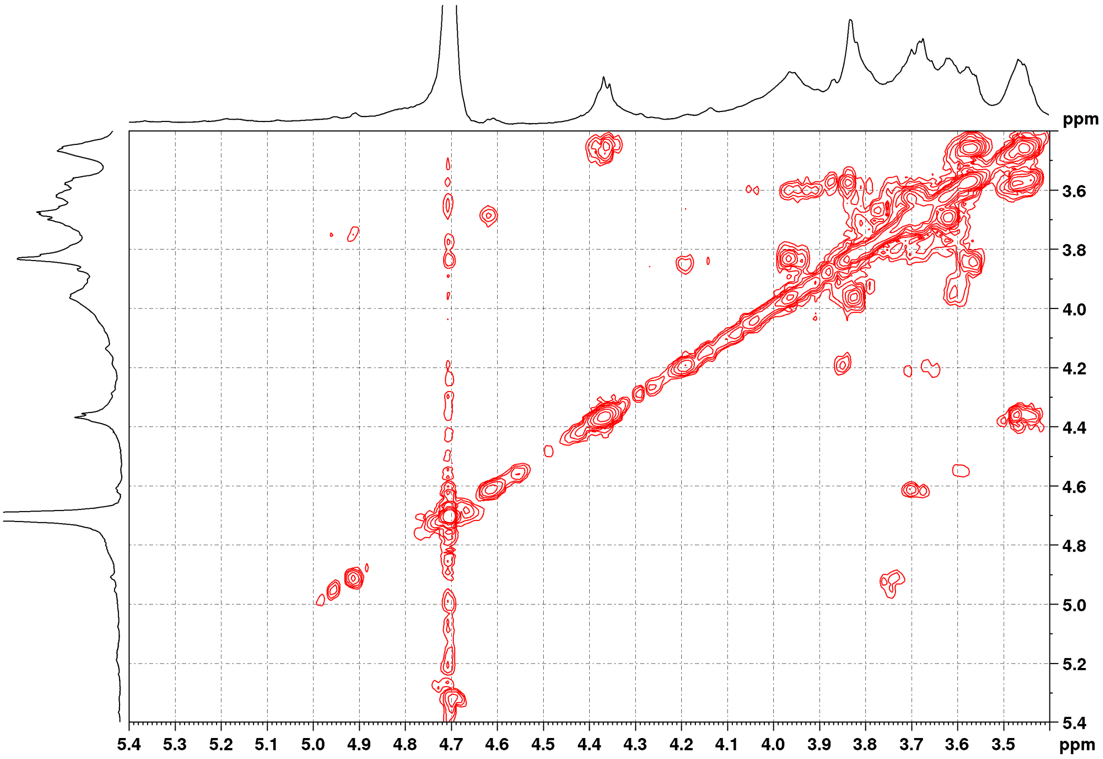

Figure S1. COSY spectrum of arabinogalactan isolated from *Larix sibirica* wood.

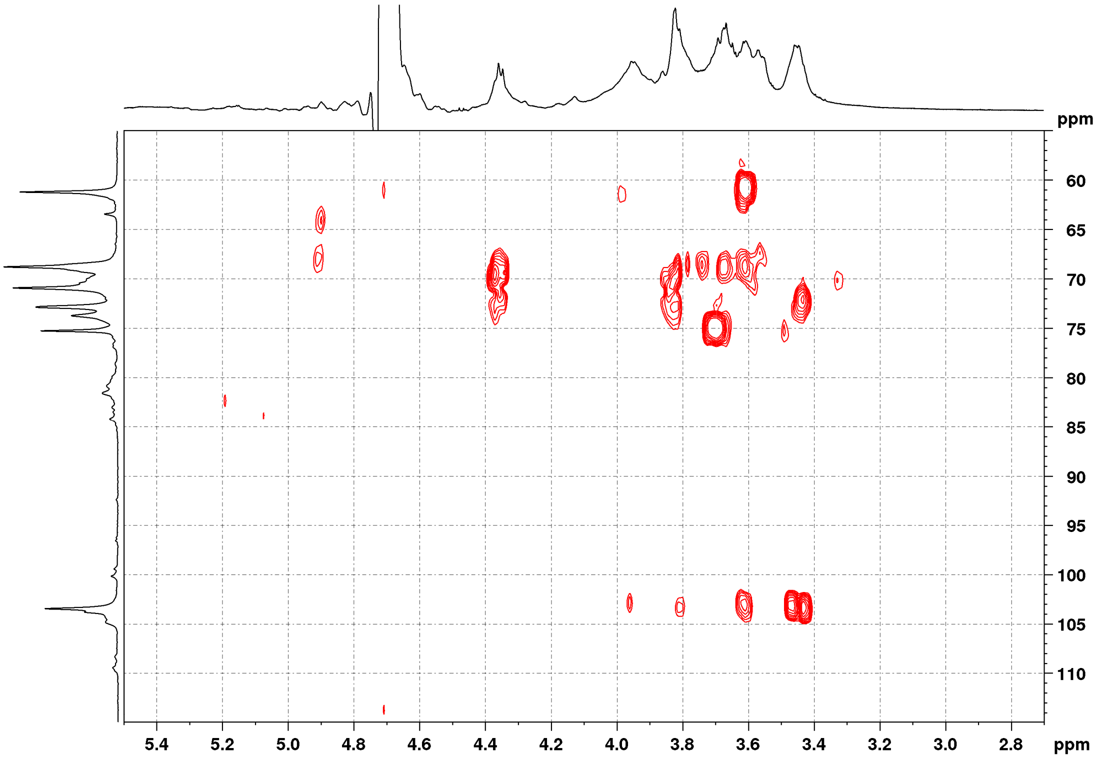

Figure S2. HMBC spectrum of arabinogalactan isolated from *Larix sibirica* wood.

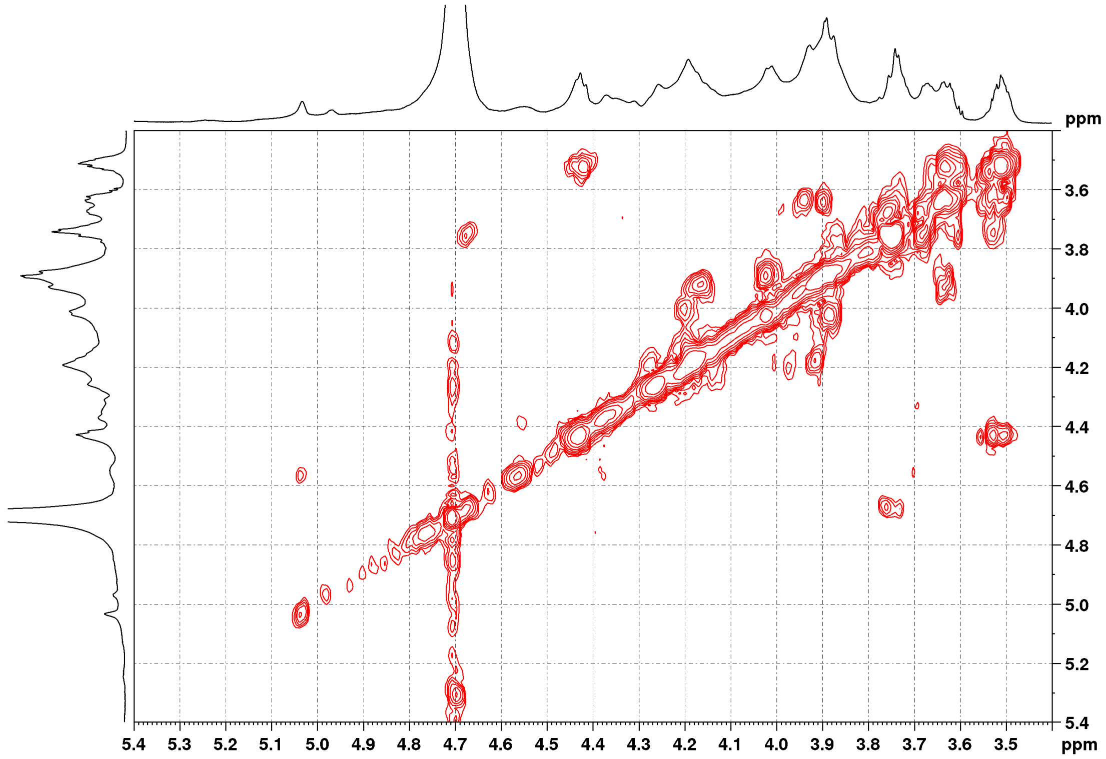

Figure S3. COSY spectrum of sulfated arabinogalactan sample (sulfur content 11.6 wt %) obtained in the melt of a sulfamic acid–urea mixture.

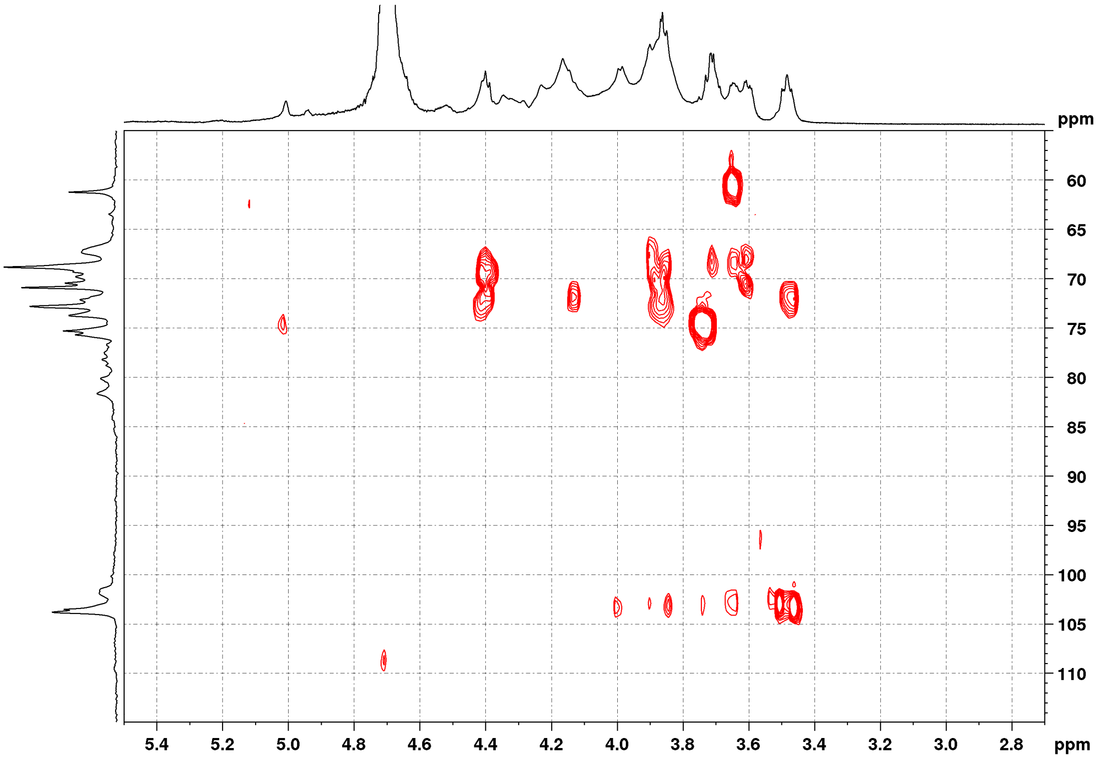

Figure S4. HMBC spectrum of sulfated arabinogalactan sample (sulfur content 11.6 wt %) obtained in the melt of a sulfamic acid–urea mixture.
